# Humans Integrate Monetary and Liquid Incentives to Motivate Cognitive Task Performance

**DOI:** 10.3389/fpsyg.2015.02037

**Published:** 2016-01-21

**Authors:** Debbie M. Yee, Marie K. Krug, Ariel Z. Allen, Todd S. Braver

**Affiliations:** Cognitive Control and Psychopathology Lab, Psychological and Brain Sciences, Washington University in St. LouisSt. Louis, MO, USA

**Keywords:** reward, motivation, cognitive control, reward integration, decision-making, subjective value, primary and secondary incentives

## Abstract

It is unequivocal that a wide variety of incentives can motivate behavior. However, few studies have explicitly examined whether and how different incentives are integrated in terms of their motivational influence. The current study examines the combined effects of monetary and liquid incentives on cognitive processing, and whether appetitive and aversive incentives have distinct influences. We introduce a novel task paradigm, in which participants perform cued task-switching for monetary rewards that vary parametrically across trials, with liquid incentives serving as post-trial performance feedback. Critically, the symbolic meaning of the liquid was held constant (indicating successful reward attainment), while liquid valence was blocked. In the first experiment, monetary rewards combined additively with appetitive liquid feedback to improve subject task performance. Aversive liquid feedback counteracted monetary reward effects in low monetary reward trials, particularly in a subset of participants who tended to avoid responding under these conditions. Self-report motivation ratings predicted behavioral performance above and beyond experimental effects. A follow-up experiment replicated the predictive power of motivation ratings even when only appetitive liquids were used, suggesting that ratings reflect idiosyncratic subjective values of, rather than categorical differences between, the liquid incentives. Together, the findings indicate an integrative relationship between primary and secondary incentives and potentially dissociable influences in modulating motivational value, while informing hypotheses regarding candidate neural mechanisms.

## Introduction

On a daily basis, humans are faced with the formidable feat of integrating multiple diverse incentives to pursue behavioral goals. However, while most extant studies of reward support the powerful role for motivational incentives in driving cognitive processing and behavior (Atkinson, 1964; Bolles, 1975; McClelland, 1987; Weiner, 1989), they rarely account for (and often ignore) how different categories of incentives motivate behavior. This is particularly true in human cognitive experiments that examine incentive effects on task performance (Camerer and Hogarth, 1999; Bonner and Sprinkle, 2002). These studies traditionally use monetary rewards as incentives, and “motivation” is often defined as the behavioral differences between high and low monetary reward conditions (e.g., a participant who responds faster and more accurately during conditions with greater monetary rewards is assumed to be more “motivated”). As such, the well-controlled laboratory studies examining monetary incentives effects on task performance tend to have a more limited view of motivation, and do not capture variation from the wide array of motivational influences that regularly drive human behavior. This is problematic as these studies may fail to capture the complexity of motivational processing that occurs in real world decision-making.

Studies that solely use monetary incentives (a secondary reward) to measure the impact of motivational incentives on task behavior neglect the role of primary incentives (e.g., food and sex) that may be more “hard-wired” in their influence on human behavior (Krug and Braver, 2014). More importantly, humans appear to seamlessly “bundle” different categories of incentives to influence behavioral decisions (e.g., deciding whether to spend $100 to treat a visiting friend to a fancy a steak dinner or finally take care of that overdue dental cleaning). While the idea of a computing a subjective value for bundled incentives is not new (Rangel et al., 2008; FitzGerald et al., 2009; Padoa-Schioppa and Cai, 2011), it is less evident how to measure how this “value computation” explicitly biases cognition and behavior. For instance, imagine an individual who works for a company that provides delicious snacks in a break room for all of their workers. This individual may be driven to perform well in order to earn their paycheck, but also by the snacks, which together enhance the value of working at that particular company. In this scenario, this worker's motivation may depend on a combination of both the salary (secondary) and available snacks at the office (primary), but it is difficult to estimate the specific contribution of each incentive to their overall motivation to work at that job.

To further complicate things, incentives can either provide a purely symbolic or a value-dependent motivational effect on goal-directed behavior (McClelland, 1987). A purely symbolic incentive will indicate the relative importance of a particular trial and/or condition, which can help modulate cognitive processing during a task. In this scenario, positive and negative incentives are equally informative, as both provide clear signals of task performance. Conversely, a value-dependent motivational incentive will both signal the importance and influence the subjective value of the task goal; accordingly, it should modulate behavioral task performance in proportion to that subjective value. Specifically, a participant's task performance will be contingent on whether a positive (i.e., appetitive) or negative (i.e., aversive) incentive is used, and moreover, how strongly they subjectively value that incentive. Monetary reward is a well-known motivational incentive, as individuals expend more effort and utilize more cognitive resources to perform better on tasks with monetary reward (Knutson et al., 2000; Small et al., 2005; Engelmann et al., 2009; Hübner and Schlösser, 2010). However, it remains highly disputed whether non-monetary incentives have similar or distinct motivational effects on cognitive task performance (Krug and Braver, 2014).

Two distinct theories have emerged that putatively explain how primary and secondary rewards combine to modulate cognitive processing and behavior. One possibility is that primary and secondary rewards are integrated in terms of their behavioral influence, but modulate decision-making via separate parallel neural mechanisms. Some fMRI neuroimaging evidence suggests that primary and secondary rewards produce similar behavioral changes in a working memory task, albeit through distinct neural mechanisms of reinforcement (Beck et al., 2010). Moreover, a meta-analysis of human functional neuroimaging studies by Sescousse et al. (2013) revealed that different categories of incentives were represented more strongly in distinct brain structures within the reward network (e.g., money elicits the orbitofrontal cortex, while foods elicit the anterior insula). Notably, this insula activity may relate to the interoceptive effects of primary incentives, which is not present with abstract secondary incentives. Conversely, some researchers argue that the subjective values of primary and secondary rewards are initially combined into a “common currency” which is then used to bias subsequent decisions (McNamara and Houston, 1986; Levy and Glimcher, 2012). This view has been bolstered by fMRI studies that illustrate that ventromedial prefrontal cortex (vmPFC) and orbitofrontal cortex (OFC) are activated during economic choices about food and monetary rewards (O'Doherty, 2007; Chib et al., 2009), as well as in anticipation of juice and monetary rewards (Kim et al., 2011). Critically, these latter studies suggest that individuals may process expected rewards without regard to category of incentive.

A related question is how humans combine appetitive and aversive incentives to modulate behavior. Studies have shown that when individuals made economic decisions about combined monetary rewards and painful shock stimulations, the subjective expected utility of the integrated incentives was correlated with neural activity in vmPFC and other value-sensitive brain regions, including ventral striatum (VS) and anterior cingulate cortex (ACC; Talmi et al., 2009; Park et al., 2011). Notably, the these brain regions are thought to be involved with integrating action costs and benefits of a decision (Botvinick et al., 2004; Croxson et al., 2009; Fujiwara et al., 2009; Shenhav et al., 2013). These studies provide evidence that humans may encode the values of diverse incentives with conflicting valence, and that this motivational conflict is not restricted to incentives of the same type. However, a limitation of these prior studies is that most have measured incentive integration in terms of preference decisions, rather than in terms of the effects of incentives in motivating goal-directed behavior (e.g., how much does an incentive impact task performance, which is more similar to real-world scenarios). To fully capture the effects of incentives on goal-directed tasks, it is necessary to empirically measure how combined primary and secondary incentives translate into quantifiable changes in cognition and behavior.

To investigate these questions, we developed a novel experimental paradigm that examines the effects of monetary and liquid incentives on goal-directed behavior, via a classic task of cognitive control: the cued task-switching paradigm. While task-switching *per se* is not the primary focus of the study, we use this paradigm since it is considered to be a hallmark task of cognitive control (Monsell and Driver, 2000) in that successful task performance relies upon the flexible maintenance and updating of multiple task-rules in working memory to select the appropriate action to successfully perform the task. In this paradigm, each trial begins with a cue to indicate which task to perform on that trial, either a letter judgment (vowel vs. consonant classification) or a number judgment (odd vs. even classification). The cued task varies randomly from trial to trial; because the target stimulus is always ambiguous (a letter-number pair), so cognitive control is required to disambiguate the target, based on the relevant task goal for that trial. As such, the task was relatively demanding, and participants would only successfully perform the task if they were motivated enough. Monetary incentives were used as a means of manipulating motivation in a symbolic manner. The monetary reward varied on a trial-by-trial basis, and was indicated with dollar sign symbols presented simultaneously with the task cues ($, $$, or $$$$; denoting that the trial had low, medium or high reward value, respectively). Reward was earned on a trial when participants performed both fast and accurately.

The key novel component is that when participants earned the monetary reward available on a trial, this successful reward attainment was signaled via a drop of either appetitive (juice), neutral (a tasteless neutral solution, or aversive (concentrated saltwater) liquid delivered directly to their mouth. Critically, since the liquid feedback only served as an informational signal about performance success in each trial, the symbolic utility of the liquid was conveyed solely by its (presence vs. absence, which indicated a failure to attain the monetary reward). We emphasized the symbolic utility of the liquid by manipulating liquid valence across blocks (rather than on a trial-by-trial basis), and only incidentally mentioning that the liquid might vary in this manner, rather than by explicitly calling attention to the manipulation (e.g., via trial-by-trial cueing, which could emphasize the different symbolic or categorical properties of the different liquids). Because of the subtle and unobtrusive nature of the liquid manipulation, we might predict that individuals would perform similarly across all task blocks regardless of the type of liquid they receive as feedback. Alternatively, if the liquid valence (positive, neutral, negative) does influence task performance under these conditions, it would demonstrate that individuals incidentally integrate the subjective motivational value of the liquid with the monetary incentive to affect behavior. The latter result would have important theoretical implications for how individuals combine multiple categories of incentives to modulate the cognitive processing in the pursuit of behavioral goals.

In the current study, we addressed three scientific questions. First, we explored whether and how primary and secondary rewards are integrated to modulate motivational and cognitive processes. If humans process primary and secondary reward mainly in terms of symbolic value, then the type of liquid given as feedback should not influence task performance. Alternatively, individuals may incorporate the subjective motivational value of the liquid feedback with monetary reward, modulating task performance accordingly. In such a case, we might predict that the appetitive value of the liquid combines additively with monetary reward value in terms of its influence on cognitive processing and task performance. While the latter result may be surprising, given the symbolic role of the liquid incentives, it would suggest that humans automatically integrate the monetary and liquid rewards into a “common currency” utility component that biases goal-directed behavior.

Second, we tested whether appetitive and aversive motivational incentives have distinct impacts on cognitive processing. If appetitive and aversive liquids were processed via the same cognitive mechanism, we should observe additive effects of both liquid types on task performance (i.e., with the appetitive liquid increasing the monetary reward value and aversive liquid reducing the monetary reward value). Alternatively, different liquid valences may have distinct motivational effects on task performance (Kahneman and Tversky, 1979). In this case, we would still predict an additive effect of two appetitive incentives (e.g., juice and money) on task performance. However, if aversive incentives combine with appetitive incentives via a separate mechanism (i.e., one that is responsible for integrating benefits with costs), this integration could result in either a net positive or net negative motivational value. In such a case, we might expect an interactive effect of the two incentive types (e.g., saltwater and money), in which the aversive liquid has a particularly strong deleterious impact on performance in trials with low monetary reward, signifying when motivational value becomes negative. In other words, the presence of an interactive, rather than additive, effect of saltwater on task performance would provide evidence for separate mechanisms responsible for aversive vs. appetitive motivational integration.

Third, we sought to provide convergent evidence that the reward manipulations utilized in this study produced value-dependent motivational effects that were highly subjective (i.e., idiosyncratic) in nature. Toward this end, we also used self-report ratings to assess participant motivation in each of the task conditions. In order to provide stronger support for the utility of self-reported motivational ratings, we also conducted a second experiment with the same task paradigm, but including only different appetitive liquid rewards (e.g., three juices), in conjunction with monetary incentives. By eliminating categorical differences across the liquids, we directly tested whether task performance reflected the subjective motivational value of the liquid incentives (as measured by self-report motivational ratings), rather than their categorical or symbolic properties.

## Experiment 1: how do appetitive and aversive liquid incentives influence motivation to attain monetary incentives?

### Materials and methods

#### Participants

Forty-two adults (27 females; ages 18–32; *M* = 20.3; *SD* = 2.4) were recruited from the Washington University Psychology Department Experimetrix Subject Pool. The study was carried out in accordance with the recommendations of the Institutional Review Board of the Human Research Protection Office at Washington University, St. Louis. All participants provided written informed consent, and were given a payment for their participation ($20 for a 2-h session), with additional earnings based on performance up to seven dollars (*M* = *$*4.80, *SD* = *$*0.93). Three participants were excluded from analyses due to experimental and/or technical error (*N* = 39; 27 females, ages 18–32, *M* = 20.3 *SD* = 2.5). Data were collected and managed using a secure web-based application, Research Electronic Data Capture (REDCap), hosted at Washington University (Harris et al., 2009).

#### Task

**Figure 1:**
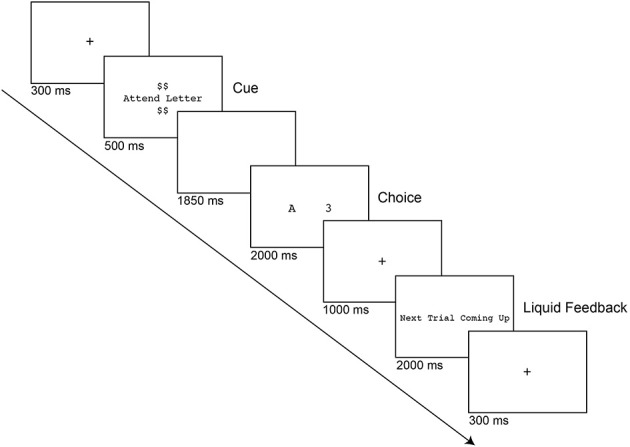
**Letter-digit task-switching paradigm**. Each trial began with a fixation cross, followed by a cue that indicated the categorization rule (e.g., “Attend Number” vs. “Attend Letter”) and the reward value of the trial, when relevant (e.g., $, $$, or $$$$). During practice and baseline trials, participants were told that the dollar signs had no importance. The target was presented after 1850 ms, which consisted of a letter and a digit displayed at the center of the screen. Participants made either a vowel/consonant or odd/even judgment depending on the cue instruction. During incentive runs, participants received 2 mL of liquid (juice, neutral, or saltwater) as feedback if they were accurate and faster than their reward criterion (determined from baseline performance. Liquids were blocked across runs and counterbalanced. Following the feedback (if applicable), a fixation cross was presented until the start of the next trial.

Subjects performed a computerized letter-digit task-switching paradigm (see Figure 1) programmed in E-Prime Version 2.0.10.242 (Psychology Software Tools, Pittsburgh PA; www.pstnet.com). Each trial began with a fixation cross for 200 ms, followed by brief fixation flicker for 100 ms to signal the upcoming cue. Next, a cue was presented for 500 ms to indicate which task to perform on that trial. If the cue text was “Attend Letter,” the task would be to classify a letter as a vowel or a consonant, whereas if it was “Attend Number,” the task would be to classify a number as odd or even. The number of dollar signs displayed above and below the cue indicated the reward value of the trial during the incentive runs (See Procedure). Following a cue-to-target interval blank screen of 1850 ms, the target stimulus was presented for up to 2000 ms, which consisted of a letter and a digit displayed in the center of the screen. Since every target stimulus contained both a letter and number, cognitive control is recruited to appropriately update the relevant task goal for that trial. Subject responses were recorded using an E-prime SR box, and response mappings were counterbalanced between participants. After the target was removed, a fixation cross appeared on the screen for 1000 ms, followed by feedback. During practice sessions, participants received written verbal feedback that was visually presented on the screen to indicate whether the trial was correct, incorrect or too slow. In baseline and incentive conditions, participants no longer received visual feedback on their performance, but simply saw the text “Next Trial Coming Up” for 2000 ms after each completed trial. In the incentive condition, participants received a squirt of liquid as feedback if they were accurate and were faster than the reward criterion that was calculated after the baseline session (37.5th percentile of correct RTs from task-switching blocks). Following the feedback, a fixation cross was presented until the start of the next trial.

#### Procedure

Participants performed three practice runs of the letter-digit task-switching paradigm. First, they practiced the letter task or digit task (12 trials each run, order was counterbalanced), in which they only saw the same written task cue for the entire run. Afterwards, they practiced task-switching, in which both “Attend Letter” and “Attend Number” task cues were intermixed during the run (24 trials). During practice runs, participants received performance feedback after each trial. The experimenter was available to answer questions and ensure that the participant understood the task.

Next, participants performed three longer baseline runs. During baseline runs, participants performed the same letter-digit task but received no performance feedback. First, they performed two single-task baseline runs (letter only or digit only, 48 trials in each block), followed by a task-switching baseline run consisting of 96 trials. Subjects were instructed to respond as quickly and accurately as possible. Participants performed with an average response time of 806 ms (*SD* = 190 ms) and with 90% accuracy (*SD* = 0.12) in the task-switching baseline blocks, suggesting both that the task was indeed challenging, but also that participants could perform it with a high-level of proficiency. Although dollar signs symbols were always presented with the task cues, in the baseline conditions participants were told that they did not hold any significance.

Afterwards, the participant performed three runs of letter-digit task-switching in the incentive condition. Participants were informed that they could earn monetary reward in that trial if they responded accurately and faster than a reward criterion. The reward criterion was calculated for each participant, based on the 37.5th percentile of correct reaction times (RT) during the task-switching baseline run. This reward criterion was set to make the incentive blocks challenging, such that the participants would have to substantially improve their performance relative to baseline (i.e., maintaining accuracy while increasing speed), in order to earn rewards on the majority of trials during the incentive runs.

If the participant was both accurate and faster than their reward criterion, they received a squirt of liquid as feedback to indicate that they had earned the monetary reward for that trial. Conversely, if they did not earn the monetary reward, they did not receive any liquid feedback, which indicated that they did not have the performance level necessary to receive the monetary reward. The number of dollar signs presented with the cue indicated how much money the participant could earn per trial (“$” = low, “$$” = medium, “$$$$” = high). While participants were not told the exact dollar amount per trial (to minimize the likelihood that they would try to maintain a running count of earned monetary reward), they understood that the dollar signs indicated the relative worth of each trial type. They could earn up to seven dollars in addition to their hourly payment, which they would receive at the end of the experiment. Reward cues and letter-digit presentation order were randomized and counterbalanced across subjects.

The incentive condition consisted of three runs, with each run containing two blocks of 48 trials (six blocks total) Each run had a different feedback liquid (apple juice, an isotonic tasteless neural solution, and concentrated saltwater), with liquid order counterbalanced between subjects. Liquid was delivered via a digital infusion pump (model SP210iw, World Precision Instruments, Inc.) and Tygon tubing directly to the participant's mouth. The liquid pump was triggered by an output signal from the E-Prime script that delivered 2 mL of liquid as feedback if participants earned the monetary reward (i.e., both accurate and faster than the reward criterion). Participants knew that the liquids changed across runs, but this manipulation was not explicitly emphasized in the instructions (i.e., liquid identity was treated as an incidental factor).

Participants filled out various self-report questionnaires upon completing the letter-digit task-switching paradigm. They rated how much they liked the three liquids, as well as how intense they were, on a 7-point Likert Scale. They also rated their motivation, performance, and how much they liked performing the low reward, medium reward, and high reward trials in each of the three liquid conditions on a 7-point scale. Following completion of the questionnaires, participants were informed of their additional earnings, paid, and debriefed.

### Results^1^

#### Incentive effects on reward rate

We first examined the effect of monetary and liquid incentives on task performance, which we quantified in terms of reward rate. Reward rate was defined as the percentage of rewarded trials in each condition of the experiment (when the subject was accurate and faster than the reward criterion). Critically, participants needed to significantly improve their task performance in order to have a high reward rate in the incentive trials; so enhanced performance reflects increased “motivation” during the incentive blocks. With a 3 × 3 repeated measures omnibus ANOVA, we observed significant effects of monetary reward [*F*_(2, 76)_ = 28.095, *p* < 0.001] and liquid feedback [*F*_(2, 76)_ = 20.102, *p* < 0.001], as well as a significant interaction [*F*_(4, 152)_ = 4.868, *p* = 0.001]. See Table 1 for reference. We next decomposed this omnibus pattern, in order to better understand the nature of the observed effects.

**Table 1:** **Reward Rate, Error Rates, and Response Times (Experiment 1)**.

			**Monetary reward**		
			**Low ($)**	**Medium ($$)**	**High ($$$$)**
**Liquid feedback**	**Juice**	*Reward rate*	0.733 (0.107)	0.741 (0.116)	0.777 (0.116)
		*Response time*	574 (60)	570 (57)	543 (52)
		*Error rate*	0.146 (0.076)	0.149 (0.074)	0.135 (0.072)
	**Neutral**	*Reward rate*	0.672 (0.112)	0.679 (0.095)	0.739 (0.091)
		*Response time*	600 (61)	596 (61)	560 (59)
		*Error rate*	0.158 (0.062)	0.151 (0.079)	0.151 (0.076)
	**Saltwater**	*Reward rate*	0.526 (0.211)	0.575 (0.124)	0.704 (0.133)
		*Response time*	670 (12)	660 (97)	599 (92)
		*Error rate*	0.199 (0.099)	0.183 (0.064)	0.148 (0.066)

*Table of reward rates, response times, and error rates by experimental condition (three levels of monetary reward and three types of liquid feedback). The mean score per experimental category is listed, with standard deviation in parentheses. Reward rate is the percentage of incentive trials for which monetary incentive was received. Subjects received a monetary reward during a trial if they were both accurate and faster than a reward criterion, which was established based on performance from the mixed baseline run. Response time includes only correctly responded trials, in milliseconds. Error rate is the percentage of commission errors (i.e., excluding no response trials)*.

#### Monetary incentives improve reward rate

To examine the “pure” effects of monetary reward, we first examined data from the neutral liquid condition only. A 3 × 1 repeated measures ANOVA tested the effect of monetary reward on reward rate. We observed a monotonic effect of monetary reward, in which increased reward rates were attained with increasing amounts of monetary reward, which was signaled by receiving the neutral solution as feedback [*F*_(2, 76)_ = 7.547, *p* = 0.001].

#### Liquid feedback also modulates reward rate

**Figure 2:**
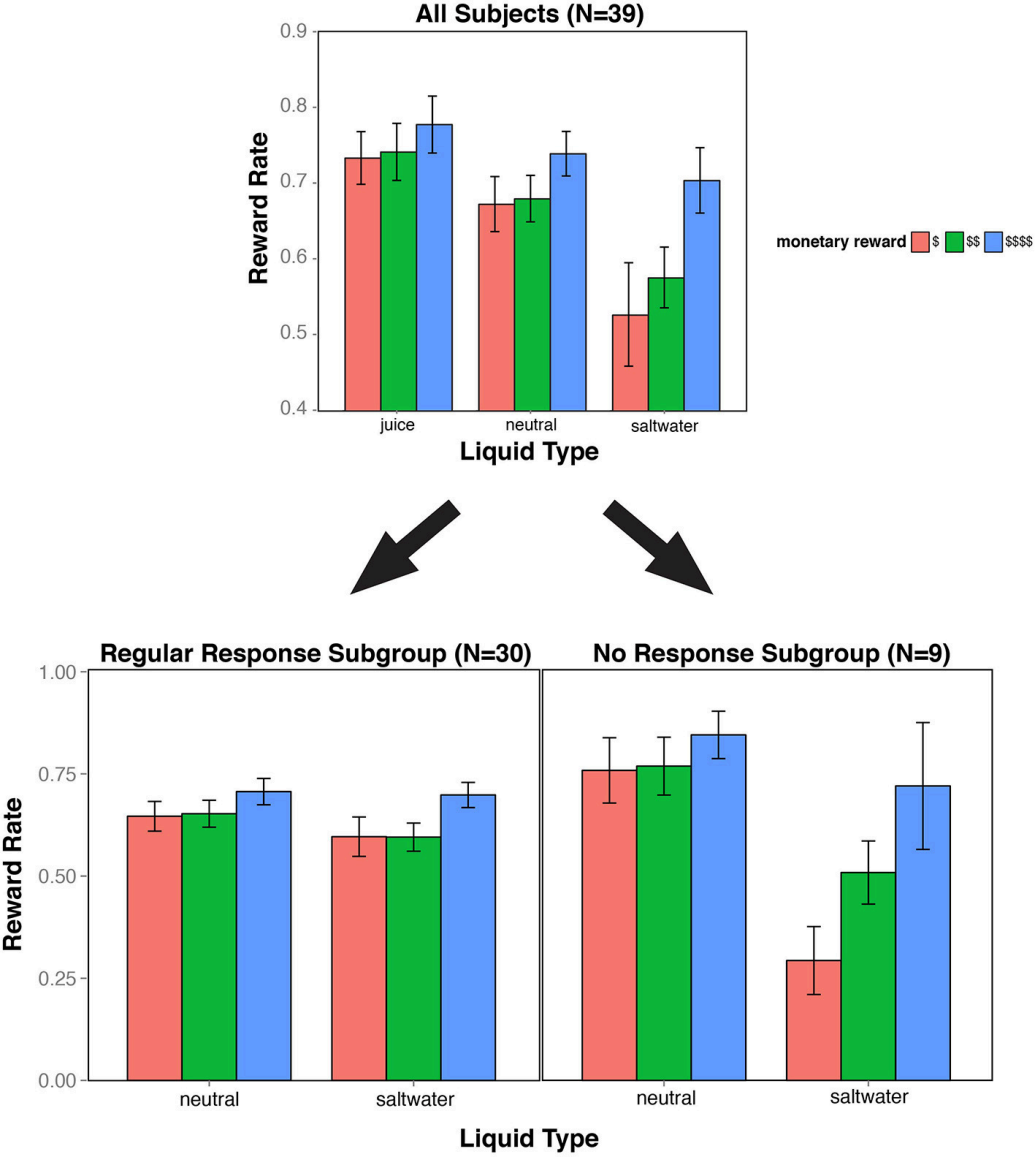
**Reward rate bar plot**. The bar plot indicates reward rate performance as a function of experimental condition (three levels of monetary reward, and three types of liquid feedback). The top figure includes all 39 subjects in the analysis. This figure highlights the significant main effects of monetary reward, liquid valence, and the interaction of the two factors when contrasting neutral and saltwater conditions. The data were further divided into two subgroups. In the No Response subgroup (below right), the interaction pattern was even more strongly present, while in the Regular Response subgroup (below left), the interaction was absent, though there was still a (trend-level) effect of saltwater on reward rate.

Next, we examined whether the type of liquid used as feedback provided additional motivational effects on reward rate beyond monetary incentive effects. We separately compared reward rate effects between juice and neutral solution conditions, as well as between saltwater and neutral solution conditions, in order to better isolate appetitive and aversive liquid valence effects. The reward rates across all monetary and liquid conditions are illustrated in Figure 2.

##### Juice vs. neutral

Participants performed better with juice than with neutral solution as liquid feedback. A 3 × 2 repeated measures ANOVA showed a main effect of liquid type [F_(1, 38)_ = 9.660, *p* = 0.004], with an average reward rate of 75% in the juice blocks, compared to 70% in the neutral blocks. The ANOVA also confirmed that the main effect of monetary reward was still present when combining both liquid types [F _(2, 76)_ = 11.288, *p* < 0.001]. Importantly, there was no hint of an interaction between the two factors [F _(2, 76)_ = 0.463, *p* = 0.631], indicating that their effects on reward rate were additive.

##### Saltwater vs. neutral

Participants had lower reward rates with saltwater than with neutral solution as liquid feedback. A 3 × 2 repeated measures ANOVA demonstrated a main effect of liquid type [F_(1, 38)_ = 15.271, *p* < 0.001], with an average reward rate of 60% in the saltwater blocks, compared to the 70% in the neutral blocks. The main effect of monetary reward was still present, with higher reward rates for greater monetary reward [F_(2, 76)_ = 25.222, *p* < 0.001]. Notably, there was a significant interaction between the monetary reward and liquid factors [F_(2, 76)_ = 4.864, *p* = 0.010]. Post hoc analyses revealed that the saltwater most strongly impacted reward rate in trials with low monetary reward. Subjects performed worse with saltwater feedback on trials with low and medium monetary reward [*t*_(38)_ = 3.570, *p* < 0.001; *t*_(38)_ = 3.830, *p* < 0.001], but not during trials with maximum monetary reward [*t*_(38)_ = 1.383, *p* = 0.175].

When we further examined the individual subject patterns in the data, we discovered that a subset of the subjects actually withheld some of their responses during low reward trials in the saltwater condition. We performed a more focused analysis to determine whether these intentional “no responses” contributed to the observed interactive pattern between saltwater and neutral solution liquid feedback.

#### No response subgroup

While most of the 39 participants responded on every task trial, a subset of nine participants withheld responses from at least three trials, exclusively during the low reward trials of the saltwater condition, with a range from 7 to 62 “no responses.” We categorized these nine participants as our “No Response” (NR) subgroup. We ran a 3 × 2 × 2 ANOVA on reward rate with monetary reward, liquid type, and group as factors. We observed significant effects of monetary reward and liquid type [*F*_(2, 74)_ = 31.825, *p* < 0.001; *F*_(1, 37)_ = 28.252, *p* < 0.001], and a trend-level effect of response subgroup [*F*_(1, 37)_ = 2.964, *p* = 0.093]. Moreover, there were significant two-way interactions between subgroup and liquid type [*F*_(2, 74)_ = 33.302, *p* < 0.001], and between subgroup and monetary reward [*F*_(1, 38)_ = 10.949, *p* < 0.001]. Most critically, we observed a significant three-way interaction between subgroup, liquid feedback, and monetary reward [*F*_(2, 74)_ = 7.686, *p* < 0.001].

Based on these findings, we analyzed the “Regular Response” subgroup separately to test whether it was the presence of the NR subgroup that drove the Two-way interaction observed in the full dataset. When we omitted the NR subjects from our analyses, we still observed an effect of monetary reward [*F*_(2, 58)_ = 16.530, *p* < 0.001], and a marginally significant effect of liquid feedback [*F*_(1, 29)_ = 3.658, *p* = 0.066]. Critically, the interaction between monetary reward and liquid feedback was no longer even close to significant [*F*_(2, 58)_ = 1.052, *p* = 0.356]. However, even in this “Regular Response” subgroup, better performance in the saltwater condition was observed on higher monetary reward trials (*M*_H_ = 0.720, *SD*_H_ = 0.085) compared to low and medium monetary reward trials (*M*_M_ = 0.617, *SD*_M_ = 0.095; *M*_L_ = 0.618, *SD*_L_ = 0.024).

These results reveal that the interactive pattern between the monetary reward and aversive liquid incentive (saltwater) observed in the full dataset was primarily driven by the “No Response” subgroup. One possibility is that these NR subjects may have been particularly sensitive to saltwater, which may have biased them to withhold responding to eliminate the risk of receiving saltwater altogether in low monetary reward trials. While the “Regular Response” subgroup did not exhibit as an extreme of a response as the NR subjects in the saltwater condition, they may have also been less motivated by the prospect of earning a monetary reward in the low-reward saltwater trials, but enough to consistently overcome the aversive taste of the saltwater feedback during these trials. The reward rates divided by subgroup are illustrated in Figure 2.

#### Response times

To better understand the motivational effects of the liquid feedback on reward rate, we analyzed participant response times (RT) on correctly responded trials. We first compared RTs between juice (appetitive) and neutral solution conditions, and then between saltwater (aversive) and neutral solution conditions. The RTs are listed in Table 1.

##### Juice vs. neutral

Both the juice and monetary rewards provided positive motivational effects on participant RT. We ran a 3 × 2 repeated measures ANOVA to examine the effects of monetary reward and juice on RT. Participants were faster when receiving juice as liquid feedback, with an average RT of 562 ms, compared to 585 ms with neutral solution [F_(1, 38)_ = 6.297, *p* = 0.016]. Participants were also faster on trials where they could earn more money [F_(2, 76)_ = 22.789, *p* < 0.001]. There was no significant interaction [F_(2, 76)_ = 0.600, *p* = 0.552].

##### Saltwater vs. neutral

The saltwater provided a negative motivational effect on participant RT. We ran a 3 × 2 repeated measures ANOVA to examine the effects of monetary reward and aversive liquid (saltwater) on RT. Participants responded slower when receiving saltwater as liquid feedback, with an average RT of 639 ms, compared to 585 ms with neutral solution [F_(1, 38)_ = 8.309, *p* = 0.006]. However, participants were still faster on trials where they could earn more money [F_(2, 76)_ = 16.910, *p* < 0.001]. There was no significant interaction [F_(2, 76)_ = 0.839, *p* = 0.436].

#### Error rates

Error rates were defined strictly in terms of commission errors (i.e., excluding no response trials from the analysis). We first compared error rates between juice (appetitive) and neutral solution conditions, and second between saltwater (aversive) and neutral solution conditions. The error rates are listed in Table 1.

##### Juice vs. neutral

Participants did not differ significantly in error rates between juice and neutral solution liquid feedback conditions. A 3 × 2 repeated measures ANOVA on error rate with monetary reward and liquid feedback as factors, and indicated similar error rates across the two liquid conditions [*F*_(1, 38)_ = 0.703, *p* = 0.401], as well as across different amounts of monetary reward [*F*_(2, 76)_ = 0.500, *p* = 0.609]. There was no significant interaction [*F*_(2, 76)_ = 0.204, *p* = 0.816].

##### Saltwater vs. neutral

Conversely, the saltwater incentive was found to modulate error rates. We ran a 3 × 2 repeated measures ANOVA to examine the effects of monetary reward and liquid feedback on participant error rate. Participants committed more errors on trials in which they could earn less monetary reward [*F*_(2, 76)_ = 3.644, *p* = 0.031]. Additionally, there was a marginally significantly liquid effect, with an average error rate of 18% on saltwater blocks, compared to 15% with neutral solution blocks [*F*_(1, 38)_ = 3.322, *p* = 0.076]. There was no significant interaction [*F*_(2, 76)_ = 2.365, *p* = 0.101].

#### Switch costs

Since switch costs are typically treated as an index of cognitive control, we examined RT switch costs (i.e., subtracting task-repeat trial RTs from task-switch trial RTs) as a specific measure of monetary and liquid effects on cognitive processing. We observed a small, but significant, switch cost when we collapsed all task conditions within each subject (*M* = 25 ms, *t*_(38)_ = 5.685, *p* < 0.001). The small magnitude of the switch cost, while significant, is unsurprising as participants were given a long cue-to-target interval to prepare for the task: such long intervals are associated reduced switch costs (Meiran, 1996; Rubin and Meiran, 2005), and thus decrease the sensitivity of this index as an indicator of cognitive control demand.

Next, we tested whether RT switch costs were significantly modulated by monetary reward and liquid incentives. These analyses were conducted using the lmerTest (Kuznetsova et al., 2015) and LME4 (Bates et al., 2015) packages in the R statistical language. We ran a linear mixed-effects model on RT switch costs with subject as a random factor, and liquid type and monetary reward as fixed factors. We dummy coded the liquids (saltwater = −1, neutral solution = 0, juice = 1) and monetary reward ($ = −1, $$ = 0, $$$$ = 1). We used the standard step-up model building approach and selected the linear mixed model with minimum Akaike Information Criterion (AIC) and Bayesian information criterion (BIC; West et al., 2015). Consistent with prior work examining incentive effects on task-switching (Aarts et al., 2010; Kleinsorge and Rinkenauer, 2012), the mean RT switch cost was lowest when monetary reward was highest [$: *M*_1_ = 28 ms, *SD*_1_ = 88 ms; $$: *M*_2_ = 39 ms, *SD*_2_ = 86 ms; $$$$: *M*_4_ = 11 ms, *SD*_4_ = 72 ms]. However, there was no main effect of liquid on switch costs or any interaction. Although the null effect of liquid on switch costs is somewhat surprising, given the effect of this variable on reward rate, the overall the small magnitude of the switch cost to begin with suggests that we may have had reduced sensitivity to detect such effects.

#### Self report ratings predict unique variance in reward rate

We conducted a hierarchical multiple regression to determine whether self-report ratings had additional predictive utility on reward rate beyond the effects of liquid type and money reward. These analyses were conducted using the lmerTest (Kuznetsova et al., 2015) and LME4 (Bates et al., 2015) packages in the R statistical language. Mathematically, this is represented as a linear mixed model with the reward rate predicted by the monetary reward *m* and the liquid feedback *l* (Equation 1). The betas represent the weights for money, liquid, and the interaction, from left to right. Dummy coding was used to label the three liquids (juice = 1, neutral solution = 0, saltwater = −1) and amount of monetary reward ($ = −1, $$ = 0, $$$$ = 1). A mixed level regression analysis was conducted, with liquid type and monetary reward as fixed effects, while treating subject as a random effect, and included correlated intercepts and slopes for the fixed factors (Barr et al., 2013; Magezi, 2015). Model selection was determined by step-up model building approach and choosing the model with the lowest AIC and BIC criterion (West et al., 2015). We observed significant effects of monetary and liquid rewards, as well a significant interaction, which confirmed our previous results.

(1) $$\begin{array}{l} \text{Reward Rate}=\;\beta_{\text{m}}\;m+\;\beta_{\text{l}}\;l+\;\beta_{\text{ml}}\;ml \end{array}$$

Next, we mean-centered the self-report liking ratings (e.g., How much did you like the $ trial with juice?) and motivation ratings (e.g., How motivated were you on a $ trial with juice?), by each subject. Reward rate performance was independently and significantly predicted by liking ratings [*F*_(1, 38)_ = 38.706, *p* < 0.001], as well as by motivation ratings [*F*_(1, 38)_ = 55.892, *p* < 0.001]. Adding liking ratings to the model (Equation 2) predicted additional variance in the reward rate [$\chi_{\left(1\right)}^{2}=11.846$, *p* < 0.001]. Furthermore, adding motivation ratings significantly improved the model (Equation 3), but the liking ratings were no longer a significant factor in predicting reward rate variance [$\chi_{\left(1\right)}^{2}=11.986$, *p* < 0.001]. Interestingly, when we reversed the order and added motivation ratings before the liking ratings, motivation ratings enhanced the model [$\chi_{\left(1\right)}^{2}=23.374$, *p* < 0.001], but adding liking ratings in addition to motivation ratings did not predict any additional variance [$\chi_{\left(1\right)}^{2}=0.458$, *p* = 0.499]. These regression patterns suggest that the variance in the reward rates explained by liking is shared with motivation, but that the motivation ratings predict additional unique variance in reward rate (See Table 2).

(2) $$\begin{array}{rcl} \text{Reward Rate} & = & \beta_{\text{m}}\;m+\beta_{\text{l}}\;l+\beta_{\text{ml}}\;ml+\beta_{\text{like}}\text{like} \end{array}$$

(3) $$\begin{array}{rcl} \text{Reward Rate} & = & \beta_{\text{m}}\;m+\;\beta_{\text{l}}\;l+\;\beta_{\text{ml}}\;ml+\beta_{\text{like}}\text{like}\\ +\;\beta_{\text{mot}}\text{motive} \end{array}$$

**Table 2:** **Hierarchical linear regression of self-report ratings on reward rate**.

**Variable**	**β**	***t***	**AIC**	**BIC**
**Step 1**			−418.8	−360.9
Money	0.0479^***^	5.985		
Liquid	0.0744^***^	5.315		
Money × Liquid	−0.033^**^	−3.170		
**Step 2**			−428.7	−366.9
Money	0.0422^***^	4.930		
Liquid	0.0350^*^	2.127		
Money × Liquid	−0.033^**^	−3.091		
Like	0.0218^***^	3.656		
**Step 3**			−438.7	−373.1
Money	0.0291^**^	3.217		
Liquid	0.0332^*^	2.249		
Money × Liquid	−0.0269*	−2.518		
Like	0.0050	0.681		
Motivation	0.0289^***^	3.987		

Results of a three-step hierarchical regression are presented, which tested whether the self-report motivation and liking ratings held predictive utility above and beyond the experimental task conditions. A mixed linear model was employed, using the lmerTest and LME4 packages in the R statistical language, with reward rate predicted by monetary reward (money) and liquid feedback (liquid) in step 1. The liking ratings were added in step 2, and the motivation ratings were added in step 3. Significance Values:

* *p* < 0.05,

** *p* < 0.001,

*** *p* < 0.001.

**Figure 3:**
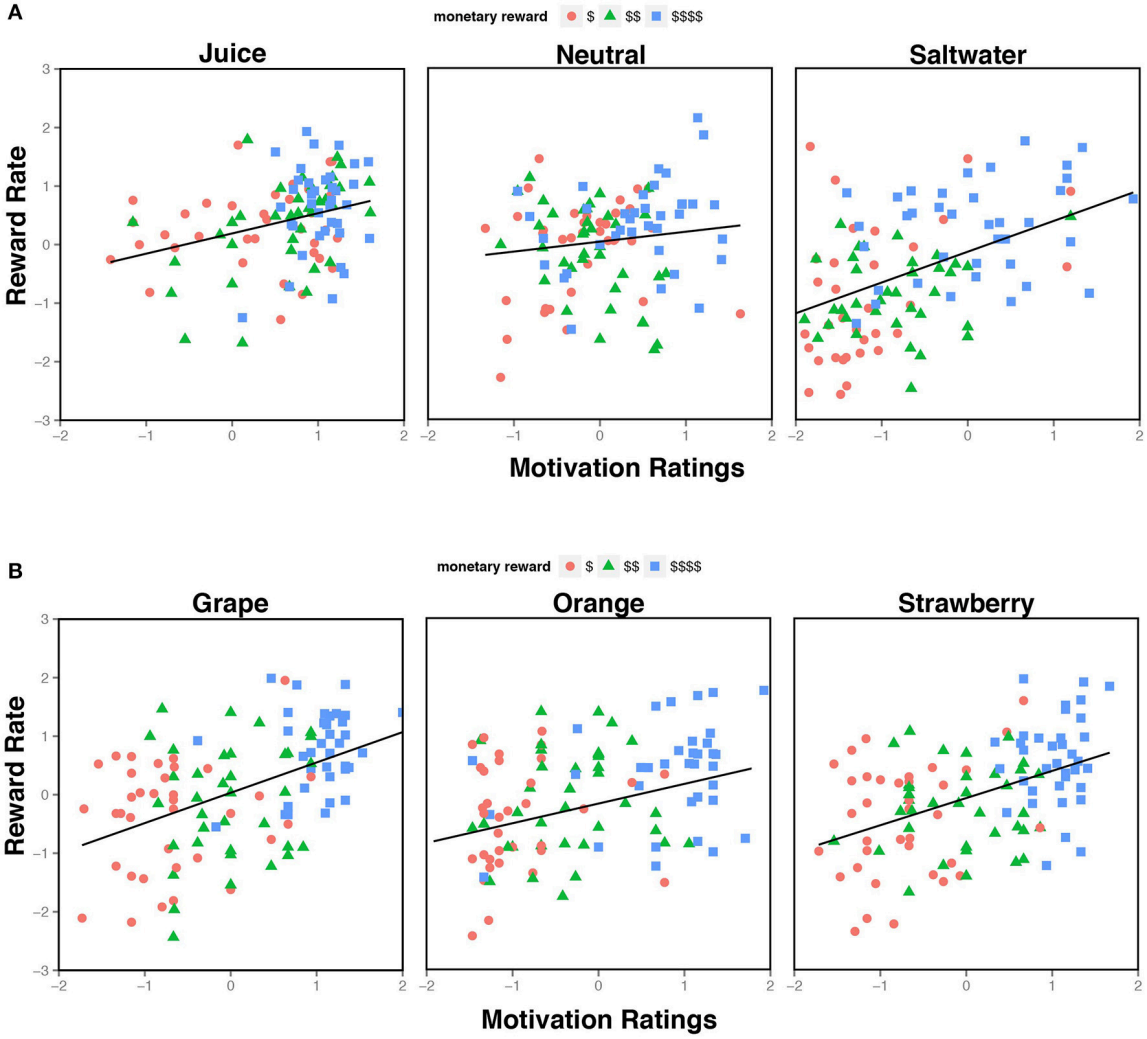
**Relationship between Motivation Ratings and Reward Rate. (A)** The scatterplot illustrates self-report motivation ratings from Experiment 1, divided by liquid feedback type, and expressed as normalized residuals (i.e., controlling for individual differences in mean rating). Each plot contains three measures (for low, medium, and high reward) for each subject. A wider spread of residuals was observed during the saltwater condition compared to the neutral and juice. **(B)** The scatterplot illustrates the normalized residuals of the motivation ratings from Experiment 2, separated by juice identity. Each plot contains three measures (for low, medium, and high reward) for each subject. Unlike the residuals in Experiment 1, there is not a clear difference in spread across the different liquid conditions.

Together, these results indicate that self-report ratings of motivation provide unique predictive utility regarding task performance across the experimental conditions, which suggests that knowing the participant's subjective motivational state can explain a significant degree of intra- and inter-individual variation in cognitive control task performance (See Figure 3A). Critically, when we removed the nine NR subjects from the dataset, these self-report ratings still significantly predicted reward rate over and above experimental task conditions. This indicates that the effects were not due to any potential anomalies (or skewed) rating profiles present in these participants. Furthermore, since motivation ratings appeared to account for unique variance over and above the liking ratings, it is possible that self-reported motivation and liking may tap into distinct psychological constructs that contribute to motivational state, such as incentive salience and hedonic value, respectively (Finlayson et al., 2007; Dai et al., 2010).

Moreover, these self-report results suggest that subjective motivational state is induced, but not completely determined, by experimental manipulations. This finding let us to conduct a second experiment, which aimed to elucidate whether the motivational state induced by monetary and liquid incentives reflects idiosyncratic subjective preferences or rather, just the categorical meaning denoted by the different liquid feedback conditions (i.e., the intrinsic properties of the liquids themselves as indicators of positive, neutral, and negative valence).

## Experiment 2: do self-report motivation ratings reflect the motivational impact of liquid incentives?

In experiment 1, the categories of liquid feedback were clearly designated (e.g., juice is appetitive, saltwater is aversive). We ran a second experiment with the same task paradigm, except using only appetitive liquids (e.g., three juices) as feedback, in order to decouple categorical meaning from subjective motivational influence across the different liquid conditions. The purpose of the second experiment was to determine whether self-report motivation ratings still predicted unique variance in task performance above and beyond the experimental reward manipulations, as in the first experiment. If so, it would provide strong evidence that these self-report ratings tap into a latent motivational state that reflects the subjective impact of each liquid (which is idiosyncratic), rather than any intrinsic (i.e., symbolic/categorical) property of the liquid incentive.

### Materials and methods

#### Participants

Thirty-nine adults (18 females; ages 18–25; *M* = 19.92; *SD* = 2.17) were recruited from the Washington University Psychology Department Experimetrix Subject Pool. The study was carried out in accordance with the recommendations of the Institutional Review Board of the Human Research Protection Office at Washington University, St. Louis. All participants provided written informed consent, and were given a payment for their participation ($25 for two separate sessions totaling 2.5 h), with additional earnings based on performance up to 7 dollars (mean = $4.90, *SD* = $0.70). One participant was excluded from analyses due to experimental and/or technical error. Similar to experiment 1, study data were collected and managed using REDCap electronic data capture tools hosted at Washington University (Harris et al., 2009).

#### Task

The task was identical to the first experiment, except that only appetitive juices were used as liquid feedback (V8 Strawberry Banana vegetable and fruit juice, V8 Tropical Orange vegetable and fruit juice, and Welch's Grape Juice) during the incentive runs.

#### Procedure

We determined the subjects' preferences for each of the three juices in a separate 15-min session prior to the task session. First, subjects participated in a preference-ranking procedure, in which the subject was presented with a choice between two of the four liquids in a single trial (three juices and one isotonic neutral solution), and asked to choose which liquid they preferred. Each possible combination of liquids was presented, and preference rankings were derived. Next, subjects tasted each of the four liquids in a random order and asked to evaluate each liquid based on its pleasantness, on a Likert scale ranging from −10 to 10. Subjects also filled out the Behavioral Inhibition/Approach System survey (Carver and White, 1994), the Generalized Reward and Punishment Expectancy Scale (Ball and Zuckerman, 1990), and the Regulatory Focus Questionnaire (Higgins and Friedman, 2001) as self-report measures of reward-related personality traits.

During the second session (on a separate day), participants performed the same letter-digit switching paradigm as in Experiment 1. They were explicitly asked to not eat or drink anything except water for 2 h prior to the start of the experiment. First, they performed three practice runs of the letter-digit task-switching paradigm, where they received performance feedback after each trial. Next, they performed three longer baseline runs, where they no longer received any performance feedback. During the task-switching baseline run of 96 trials, participants performed with an average response time of 834 ms (*SD* = 179 ms) and 92% accuracy (*SD* = 0.07). As in Experiment 1, the dollar signs were present with the task cue, but they did not hold any significance in the practice or baseline runs.

Next, the participants performed three runs of mixed letter-digit task in the incentive condition. The juice identity was blocked and counterbalanced across subjects. With the exception of the liquids used as feedback, the task procedure was identical to Experiment 1.

Participants filled out various questionnaires upon completion of the task, which included ratings that asked on a 7-point Likert scale how much they liked and how intense they found each of the three juices. They rated their motivation for performing each the low, medium, and high reward trials on each of the three liquid conditions on a 7-point scale. Lastly, they evaluated each liquid based on its pleasantness again on a Likert scale from −10 to 10. Following completion of the questionnaires, participants were informed of their additional earnings, paid and debriefed.

### Results

### Juice preference patterns

First, we examined juice preference patterns, which were derived from the preference-ranking procedure. Only 22 of the 39 total subjects demonstrated consistent (i.e., transitive) preferences. Within this subset, eight preferred grape juice as their top choice, while 10 preferred tropical orange, and one preferred strawberry banana. A 3 × 1 repeated measures ANOVA revealed that preference rank was not a significant predictor of reward rate [*F*_(2, 42)_ = 1.438, *p* = 0.248]. We ran a 3 × 1 repeated measures ANOVA on liking ratings with juice identity as a factor, and found no significant differences in liking ratings across different juice identities [*F*_(2, 74)_ = 1.835, *p* = 0.167]. Since the contrast between the liquids was less stark compared to Experiment 1, we suspected that participants tended to like all of the juices relatively equally, and may not have had strong preferences between them. This is clearly reflected in the juice Likert ratings (range 1–7), which demonstrate that individuals generally gave the juices similar ratings (M_grape_ = 4.45, M_orange_ = 4.54, M_strawberry_ = 3.95). When we more closely examined the rank-preference data, we found that 2 subjects preferred the neutral solution to all the juices, which is surprising since the juices are typically treated as appetitive incentives. Given the high rate of intransitive and inconsistent ratings, we chose not to include preference rankings in our primary analyses. However, while the rank preferences may not have been fine grained enough to detect juice preferences, we suspected that the self-report motivation ratings may have been more sensitive to the subtle influences of the juices in motivating cognitive task performance.

### Monetary reward influences task performance

Next, we looked at reward rates, separated by monetary reward amount (low, medium, high) and juice identity (e.g., grape, strawberry, or orange). A 3 × 3 repeated measures ANOVA on reward rate with monetary reward and juice identity as factors revealed a significant effect of monetary reward amount on reward rate [*F*_(2, 74)_ = 27.407, *p* < 0.001], indicating the reward rate monotonically increased with monetary reward magnitude. Juice identity did not have a significant effect on reward rate [*F*_(2, 74)_ = 1.397, *p* = 0.254], and there was no significant interaction [*F*_(4, 148)_ = 0.332, *p* = 0.865]. Performance profiles across task conditions are summarized in Table 3.

**Table 3:** **Reward Rate, Error Rates, and Response Times (Experiment 2)**.

			**Monetary reward**		
			**Low ($)**	**Medium ($$)**	**High ($$$$)**
**Liquid feedback**	**Orange**	*Reward rate*	0.661 (0.131)	0.687 (0.121)	0.750 (0.096)
		*Response time*	566 (43)	567 (65)	581 (55)
		*Error rate*	0.169 (0.063)	0.150 (0.066)	0.157 (0.071)
	**Strawberry**	*Reward rate*	0.648 (0.132)	0.678 (0.081)	0.788 (0.096)
		*Response time*	589 (82)	568 (50)	551 (48)
		*Error rate*	0.182 (0.081)	0.163 (0.062)	0.146 (0.059)
	**Grape**	*Reward rate*	0.668 (0.101)	0.711 (0.102)	0.788 (0.093)
		*Response time*	588 (66)	562 (51)	558 (69)
		*Error rate*	0.178 (0.081)	0.160 (0.069)	0.121 (0.064)

*Table of reward rates, response times, and error rates by experimental condition (three levels of monetary reward and three types of juice feedback). The mean score per experimental category is listed, with standard deviation in parentheses. Reward rate is the percentage of incentive trials for which monetary incentive was received. Subjects received a monetary reward during a trial if they were both accurate and faster than a reward criterion, which was established based on performance from the mixed baseline run. Response time includes only correctly responded trials, in milliseconds. Error rate is the percentage of commission errors (i.e., excluding no response trials)*.

### Monetary reward influences switch costs

Based on the results from Experiment 1, we also tested switch cost effects and their modulation by monetary and liquid incentives. Consistent with the Experiment 1 results, a small, but significant, switch cost was present when we collapsed all task conditions within each subject [*M* = 19 ms, *t*_(37)_ = 5.038, *p* < 0.001]. A linear mixed-effects model on RT switch costs again yielded a main effect of monetary reward [*F*_(1, 342)_ = 11.213, β = −8.041, *p* < 0.001], but no liquid effect or its interaction. Again, switch costs were lowest in the condition with the highest monetary reward [$: *M*_1_ = 21 ms, *SD*_1_ = 70 ms; $$: *M*_2_ = 31 ms, *SD*_2_ = 82 ms; $$$$: *M*_4_ = 4 ms, *SD*_4_ = 73 ms].

### Motivation ratings predict unique variance for appetitive liquids

We conducted a hierarchical multiple regression to test whether self-report motivation ratings had predictive utility on reward rate performance beyond experimental task manipulations (similar to Experiment 1). The analyses were conducted using lmerTest (Kuznetsova et al., 2015) and LME4 (Bates et al., 2015) packages in the R statistical language. We used a linear mixed model with reward rate predicted by monetary reward *m* and juice identity *j*, with beta weights for money, juice identity, and the interaction (Equation 1). Monetary rewarded was dummy coded ($ = −1, $$ = 0, $$$$ = 1), and the juice identity was effects coded (contrast 1 = grape vs. orange, contrast 2 = grape vs. strawberry). We conducted a mixed level regression with monetary reward and juice identity as fixed effects, subject as a random effect, and correlated intercepts and slopes for the fixed factors. Unsurprisingly, we observed a significant effect of monetary reward on reward rate, but no effect for juice identity.

(4) $$\begin{array}{l} \text{Reward Rate}=\;\beta_{\text{m}}\;m+\beta_{\text{j}}\;j+\beta_{\text{mj}}\;mj \end{array}$$

Next, we added motivation ratings (e.g., How motivated were you on the $ trial with Juice 1?) to the mixed model, which were mean-centered by subject. Reward rate performance was significantly predicted by motivation ratings when included as the only predictor [*F*_(1, 37)_ = 33.277, *p* < 0.001]. Moreover, when we added the motivation ratings to the model that included money and juice predictors (Equation 2), we still found that the motivation ratings predicted additional variation in reward rate [$\chi_{\left(1\right)}^{2}=20.566$, *p* < 0.001]. The betas and t values are shown in Table 4.

(5) $$\begin{array}{l} \text{Reward Rate}=\beta_{\text{m}}\;m+\beta_{\text{j}}\;j+\beta_{\text{mj}}\;mj+\beta_{\text{mot}}\text{motive} \end{array}$$

**Table 4:** **Hierarchical linear regression of motivation ratings in Experiment 2**.

**Variable**	**β**	***t***	**AIC**	**BIC**
**Step 1**			−456.7	−359.1
Money	0.0516^***^	5.291		
Juice Identity				
C1: Orange-Grape	0.0186	−1.214		
C2: Strawberry-Grape	0.0189	−1.340		
Money × C1	0.0173	−0.801		
Money × C2	0.0137	−0.489		
**Step 2**			−471.9	−363.0
Money	0.0289^*^	2.559		
Juice Identity				
C1: Orange-Grape	−0.0124	−0.733		
C2: Strawberry-Grape	−0.0244	−1.335		
Money × C1	−0.0148	−0.869		
Money × C2	−.0072	−0.525		
Motivation	0.0324^***^	5.025		

Results of a two-step hierarchical regression are presented, which tested whether the self-report motivation ratings demonstrated predictive utility above experimental task conditions, when three liquids with the same valence (appetitive) were used. A mixed linear model was employed, using the lmer Test and LME4 packages in the R statistical language, with reward rate predicted by monetary reward (dummy coded) and juice identity (contrast coded) in step 1. Motivation ratings were added at step 2. Significance Values:

* *p* < 0.05,

** *p* < 0.001,

*** *p* < 0.001.

Similar to Experiment 1, the motivation ratings significantly predicted performance above reward effects in the task (see Figure 3B). Notably the effect of monetary reward was weaker, but still significant when the motivation ratings were added, indicating that these ratings explained a large portion of the task variance. Critically, since all three liquids were juices (i.e., there was no categorical distinction between the incentives), these ratings likely reflect the subjective utility assigned to the task condition, which is idiosyncratic and unrelated to the intrinsic, categorical properties of the liquids themselves. Furthermore, these data provide strong evidence for the motivational impact of liquid incentives on task performance, which informs our understanding of how latent motivational states are explicitly linked to cognitive processing.

### Personality measures and task performance

Next, we tested whether individual difference personality measures predicted reward rate. We utilized linear regression statistical models to test whether individual differences between personality measures contributed to additional variance in reward rate. All personality measures were grand mean centered. None of these personality measures significantly predicted task performance (all *p's* > 0.2).

### Satiation effects: evidence of outcome devaluation?

Since participants received appetitive liquid on every rewarded trial in the experiment, it is possible that satiation may have been a factor influencing cognitive task performance. If present, satiation would predict reduced benefits of receiving juice as liquid feedback in later trials in the experiment (when satiation should be higher). To test putative satiation effects, we used a mixed effects logistic regression with the LME4 package (Bates et al., 2015) in the R statistical language, since this approach enables greater flexibility in modeling effects of experimental factors on a trial-by-trial basis. Our logistic regression modeled whether the log of the odds of a trial being rewarded (1 = yes, 0 = no) was predicted by monetary reward and trial order across the entire session (trials 1–288). Monetary reward was dummy coded, as in previous linear mixed models. We observed a significant negative effect of trial order [β = −0.0009, *z* = −3.238, *p* = 0.001] on the likelihood of reward attainment, such that later trials were significantly less likely to be rewarded. We replicated the same analysis for Experiment 1, but here we found no significant trial order effect. While our interpretation of these trial order effects are only speculative, they are consistent with the hypothesis that increased satiation drove participant to devalue the subjective value of the juice progressively throughout the experiment. Such effects would not be predicted to be as strong in the first experiment, given the strong categorical and valence-related differences between the liquid types. However, since these are *post-hoc* analyses, further study is warranted to more systematically manipulate and test for satiation influences on task performance.

## Discussion

Understanding how diverse types of incentives impact motivation and behavior is important, as there is much ecological validity of incentive integration in the real world. This question is particularly relevant to understanding daily decision-making or mechanisms underlying eating disorders and affective-motivation disorders, such as apathy and anergia (Kringelbach and Radcliffe, 2005; Berridge, 2009; Stice et al., 2009). Our novel paradigm provides increased experimental and interpretational leverage, by providing a clear-cut measure of how the motivational impact of primary liquid incentives can modulate monetary reward effects on cognitive performance. In particular, this paradigm provides a novel means of less-obtrusively incorporating primary incentives into experimental designs, which has great utility for further investigating the modulatory effects of a wide range of motivational manipulations on decision-making and task behaviors. Next, we discuss some of the key implications and interpretations of our primary findings.

### Integration effects of reward incentives on cognitive task performance

The results provide a clear-cut answer to our first scientific question: individuals do integrate primary and secondary rewards to modulate motivational and cognitive processes, as evidenced by the additive effect of the appetitive incentives on reward rate (i.e., juice feedback increased reward rate). When we parsed reward rate into its subcomponents (RT and error rates) in Experiment 1, we found that subjects performed faster when they earned more monetary rewards and received juice as liquid feedback, but did not commit more errors across task conditions. In other words, the rewards systematically modulated RT, but not via a speed-accuracy tradeoff. On the other hand, the fact that incentives did not reduce error rates may seem surprising, but it is possible that the lack of feedback on trials that were not accurate or fast enough may have decreased the salience of commission errors in juice blocks. Under such no-feedback conditions, it appears that the primary impact of incentives is that of increasing *response vigor*. This response vigor effect could reflect a direct impact of motivational influences on behavioral responses (Talmi et al., 2008). According to the Pavlovian-Instrumental Transfer account, appetitive rewards can induce a generalized approach-related motivational state, which exerts a concomitant influence on goal-directed action selection. Such approach-related states are associated with a host of motivationally-driven and species specific behaviors, of which increased response vigor is a well-established phenomenon (Niv et al., 2006). There is some evidence that these motivational states may arise from increased tonic levels of the neurotransmitter dopamine (DA; Niv et al., 2007; Beeler et al., 2010; Beierholm et al., 2013). This tonic feature of DA is crucial, as motivation often needs to be sustained over a long period of time during goal pursuit (e.g., going to the gym to exercise and lose weight). Thus, DA may play a key role in translating the motivational value of rewarding incentives to enhance cognitive function, when pursuing temporally-extended behavioral goals. However, this relationship remains to be elucidated, and cannot be answered within the scope of this study.

### Integration effects of aversive incentives on cognitive task performance

Addressing our second scientific question, aversive primary incentives appeared to have a distinct effect from that of primary rewards on cognitive task behavior. Specifically, aversive incentives exerted an inhibitory effect on behavior, in which participants produced increased error rates in addition to slower RTs during the saltwater block in Experiment 1. Some researchers argue that aversely motivated behavioral inhibition (curtailing of ongoing actions in light of predictions of aversive outcomes) is linked to increased levels of serotonin (5-HT; Dayan and Huys, 2008; Crockett et al., 2012). However, the research on processing aversive incentives is still sparse and relatively nascent, so more work needs to be done to establish a putative neural mechanism that may underlie how aversive motivation impacts cognition. Furthermore, one important distinction with the current study is that aversive motivation is traditionally measured more instrumentally (i.e., a certain action needs to be correct/fast enough in order to avoid a punishment). Contrasted with the current manipulation in which participants received saltwater when fast and accurate, it is possible that motivation to avoid a punishing incentive may differ from motivation to endure a punishing incentive to earn a reward. Recent evidence has found distinct neural processing of the same incentive with opposing actions (go vs. no-go), revealing an asymmetry between the computations for actions and valence (Guitart-Masip et al., 2011). Therefore, we predict that altering the structure of the task to mimic more instrumental tasks (e.g., performing fast and accurately to avoid saltwater feedback) would increase reward rate, but reduce the interaction between monetary and liquid incentives. However, this manipulation would need to be empirically tested to examine whether the impact of aversive motivation is modulated by how the aversive incentives are delivered.

While initially it appears that appetitive and aversive incentives have distinct impacts on cognitive processing (e.g., additive effect of juice, interactive effect of saltwater), the discovery of the no-response subset of subjects (*N* = 9) revealed that the interaction between saltwater and money may be driven by individual differences. Within this subset of nine subjects, the disutility of the saltwater appeared to be greater than the positive utility of low monetary rewards, which drove them to intermittently forgo the opportunity to earn a monetary reward rather than risk receiving saltwater in those trials. One plausible explanation is that these subjects might be especially reactive to saltwater, or to punishments in general. This could have led to the adoption of a more extreme behavioral inhibition strategy, that of withholding responses altogether. A previous study had found that participants with high punishment-sensitivity showed an overall increase in RT in a cognitive control task after receiving punishments (Braem et al., 2013a). However, while it is plausible that this extreme impairment of task performance may have been linked to punishment-sensitivity, we were unable to directly test this hypothesis, since we did not administer any surveys that measured reward and punishment sensitivity in Experiment 1. Taken together, these results suggest another dimension to our second scientific question regarding the relationship between appetitive and aversive incentives. Specifically, it seems that aversive incentives, under certain conditions, can exert a multiplicative impact (interaction effect) on cognitive processing, and that the strength of this interaction may be modulated by how reactive individuals are to aversive incentives. In particular, a putative hypothesis is that this interactive pattern demonstrates a qualitative shift in cognitive strategy, from approach to avoidance-driven, when the net motivational reward value of the trial shifts from positive to negative.

### Neural mechanisms enabling integration primary and secondary incentives

These results naturally raise the broader question of how the brain integrates diverse types of incentives (e.g., combining primary and secondary) to motivate cognitive processing and behavior. While our study did not involve a direct examination of neural data, the findings suggest that the present experimental paradigm might also be a potentially productive one for examining the issue of incentive integration in the brain. There has been much dispute regarding whether and how incentives are integrated into a motivational value signal that biases action planning and decision-making. One promising hypothesis is that individuals integrate the values of diverse rewards into an internal “common currency,” which is used to facilitate comparisons between future potential actions and/or rewards in order to bias cognitive processing and subsequent decisions (e.g., choice preference, task-oriented behavior; Levy and Glimcher, 2012). Prior research has suggested that vmPFC, OFC, and VS serve as potential “integration hubs,” as these brain regions have been found to respond to both primary and secondary incentives (Knutson et al., 2001; Montague and Berns, 2002; O'Doherty et al., 2002; McClure et al., 2004; O'Doherty, 2007). Moreover, there is some evidence that signals from these putative hubs correlate with activity in dorsolateral prefrontal cortex, a brain region central to higher-order cognitive processing (Hare et al., 2009).

When integrating incentives of opposing valence (e.g., conflicting motivations), we also predict attenuated activity in the ACC, a brain region involved in monitoring neural signals from conflicting sources in order to output a unified signal that modulates cognitive control (Botvinick et al., 2004; Engelmann et al., 2009). Additionally, some accounts highlight the ACC as playing an important role in integrating rewards and punishments in order to generate a “motivational” or “energizing” signal that contributes to cognitive control (Fujiwara et al., 2009). Others have argued that ACC plays a critical role as a computational substrate involved in value integration (Plassmann et al., 2010; Park et al., 2011). From this, we speculate that ACC may play a central role in integrating incentives with conflicting valence to modulate cognitive processing of task goals, but whether this integration extends to situations involving different categories of incentive has not yet been empirically tested.

We argue that investigating neural mechanisms can inform the cognitive processes that underlie incentive integration and cognitive control, and provide insight to patterns in observed task-oriented behavior. However, these questions of incentive integration and motivated cognitive control are still in its nascent stages of development, and need to be further explored in experimental work, in order to garner a more sophisticated understanding of motivation-cognition interactions.

### Motivation and cognitive control

It is intuitive to imagine that cognitive control and motivation are two distinct but intertwined drives that bias decision-making. Cognitive control refers to the processes involved in regulating cognition and actions based on currently maintained goals, while motivation (as we have operationalized it), modulates the vigor of response with which one performs that action. From this, it seems reasonable to conclude that motivational influences should impact the regulation of cognition and actions, while an individual is maintaining a single or multiple goal(s). Task performance is generally slower and more error-prone on task-switching blocks relative to single-task blocks (i.e., mixing costs), and on task-switch trials relative to task-repeat trials (i.e., switch costs; Monsell, 2003). However, individuals are also able to reduce their mixing and switch costs if they are able to adequately prepare for the upcoming task. Motivational incentives, on the other hand, can modulate cognitive control by enhancing context-sensitivity to rewards, and by increasing flexibility in response times (Aarts et al., 2011; Shen and Chun, 2011; Kleinsorge and Rinkenauer, 2012; Braem et al., 2013b; Bugg and Braver, 2015).

Consistent with these prior findings, we observed (in both experiments) that switch costs were significantly reduced when monetary rewards were highest. On the other hand, liquid incentives did not produce any modulation of switch costs, which may seem surprising given their effects on overall reward rate and general task performance (RT and error rates; Experiment 1). Nevertheless, even for monetary rewards the obtained patterns were fairly subtle and switch costs levels were low overall. The subtle pattern of observed switch cost effects was not overly surprising, given that high switch costs are thought to reflect suboptimal preparation for the upcoming task, and in the current experiment ample preparation time was provided (1800 ms cue-to-target interval). For the purposes of our study, we assumed that reward rate improvements were due to enhanced cognitive control, but this may not have been reflected in terms of a strong modulation of switch costs. It is possible that rewards may have more strongly modulated mixing costs, but we cannot confirm this since the paradigm was not designed to measure mixing costs (i.e., we did not include single-task conditions). In short, our task design was not optimized for isolating reward effects on cognitive control, as we did not explicitly manipulate switch costs or mixing costs. However, this is a possible future direction in follow up studies, such as by manipulating preparation time or by comparing with incentive effects observed in single task blocks.

### Incentives modulate motivational state to bias cognitive task performance

Addressing our third scientific question, one of the most promising results of this study was the finding that self-reported motivation ratings demonstrated utility in predicting task performance above and beyond experimental task manipulations. Since our second experiment validated that these ratings reflected subjective idiosyncratic preferences rather than intrinsic properties of the liquids themselves (e.g., symbolic/categorical knowledge that juice is good, saltwater is bad), we are confident that these ratings are truly motivational in nature. For that reason, we believe that the current results provide strong support that monetary and liquid incentives are influencing cognitive task performance via a direct change in motivational state. In other words, the observed incentive effects cannot be fully explained by a simple shift in task strategy in response to the symbolically-cued importance of each trial (i.e., $ = low importance; $$$$ = high importance), since such an account could neither explain the effects of liquid valence in Experiment 1, nor the idiosyncratic patterns of motivation ratings on task performance observed in both experiments.

However, one limitation of these self-report ratings is that they were collected at the end of the study, so they might reflect retrospective memory of motivation, as opposed to an on-line estimate of motivational state during the task. While we are confident that these ratings do reflect some level of motivation, this potential confound cannot be easily circumvented, and we acknowledge that the distinction between current and past evaluation of motivation is difficult to determine with the current measure. Despite the limited temporal resolution of these ratings, our results suggest that participants appear to have explicit access to their own motivational state, and can report this state in a distinct manner from the subjective liking of the liquid or available monetary rewards. These findings suggest the utility of probing self-reported motivation in cognitive experiments, in order to increase explanatory and predictive power regarding associated behavioral performance effects, and likewise to uncover the particular mechanisms that subserve such effects.

Both a potential limitation, but also an advantage, of using consummatory incentives is that they have the potential to induce satiation (a motivational state), which may incidentally cause individuals to devalue the subjective utility of particular actions or trials in a cognitive task. For example, a thirsty individual might perform differently in this study compared to one that had just drank a glass of water. This is particularly relevant to Experiment 2, in which we found that participants performed significantly worse (i.e., achieved lower reward rates) on later trials of the task. These trial order effects may have reflected increased satiation from earning too many juice rewards, which was not a significant factor in Experiment 1. Nevertheless, although satiation is a plausible account and potential confound present in Experiment 2, more work would be needed to further explore and understand its effects. On the other hand, the possibility of satiation-related influences on motivation and task performance could also be construed as an advantageous feature of the task paradigm. Specifically, the presence of clear outcome devaluation effects (e.g., in response to selective satiation) has been traditionally used as a diagnostic signature of the degree to which behavior is under goal-directed vs. habitual control (Dickinson and Balleine, 2002; Niv et al., 2006). Thus, stronger demonstrations of satiation related effects in the current paradigm could provide insight to the specific (and potentially distinct) mechanisms by which liquid and monetary incentives influence motivational states to modulate cognitive processing.

## Conclusion

Overall, our study provides an important first stage of evidence regarding how humans integrate primary and secondary incentives, while demonstrating that this integrated incentive signal has a strong motivational impact on cognitive task performance. The experimentally-induced motivational state changes were robustly evidenced and indexed by the predictive utility of the self-report motivational ratings, which explained significant variance in the effects of experimental task manipulations in both of the experiments. Taken together, the findings highlight the productive utility of the novel task paradigm we have developed here for investigating mechanisms of motivation-cognition interaction. These can now be further extended in studies utilizing neuroscience-based methods and neural measures (e.g., fMRI and BOLD activation).

## Author contributions

DY Involved in analysis and interpretation of behavioral data, as well as primary author on the manuscript. MK Involved in experimental design, script programming and setup for data collection. Had critical role in developing the task and ensuring feasibility of the task for testing the main hypothesis. AA Involved in data collection, as well as analysis of the motivation ratings to predict cognitive task performance. TB Involved in experimental design, guidance on data collection/analysis, understanding and interpretation of behavioral results, and contributed to writing manuscript.

## Funding

This work was funded by the National Institutes of Health, grant numbers R21 MH097260 (Motivational state as a mechanism of cognitive self-regulation) and R21 MH105800 (Neuroeconomics of cognitive effort).

### Conflict of interest statement

The authors declare that the research was conducted in the absence of any commercial or financial relationships that could be construed as a potential conflict of interest.

## Footnotes

^1^Relevant material for this paper can be accessed via Open Science Framework at https://osf.io/ajgp3/. This repository contains the raw data for Experiments 1 and 2 as well as a summary document detailing all of the analyses conducted by D.M.Y. for the manuscript, using the R statistical language, and summarized via R Markdown. This document should provide the code needed to reproduce the relevant results contained within the manuscript for each experiment. Please note that this material has not been peer-reviewed.
